# Application of auriculotemporal nerve block and dextrose prolotherapy in exercise therapy of TMJ closed lock in adolescents and young adults

**DOI:** 10.1186/s13005-021-00261-7

**Published:** 2021-03-27

**Authors:** Hongzhi Zhou, Yang Xue, Ping Liu

**Affiliations:** 1 State Key Laboratory of Military Stomatology, National Clinical Research Center for Oral Diseases, Shaanxi Clinical Research Center for Oral Diseases, 145# Western Changle Road, Xi’an, 710032 P.R. China; 2 Department of Oral and Maxillofacial Surgery, School of Stomatology, The Fourth Military Medical University (FMMU), Xi’an, 710032 China

**Keywords:** Temporomandibular joint, Closed lock, Disc displacement without reduction, Degenerative joint disease, Exercise therapy, Hypertonic dextrose prolotherapy

## Abstract

**Background:**

Temporomandibular joint (TMJ) ‘closed lock’ is a clinical condition causing TMJ pain and limited mouth opening (painful locking). Recent studies suggest an increasing prevalence of degenerative joint disease associated with the onset of TMJ closed lock in adolescents and young adults. Early interventions are recommended, but the curative effect of standard therapies remains controversial. In this retrospective study, an alternative method of non-surgical treatment of TMJ closed lock is presented, and its long-term efficacy has been observed.

**Methods:**

Forty adolescents and young adults, aged 16 to 30 years old, with distinct combination of symptoms of TMJ closed lock, were enrolled. Patients received anesthetic blockages of the auriculotemporal nerve, then performed mandibular condylar movement exercise for 10 min, and subsequently received hypertonic dextrose prolotherapy in retro-discal area of TMJ. Clinical assessments at baseline and at follow-up (2 weeks, 2 months, 6 months, and 5 years) included intensity and frequency of TMJ pain, mandibular range of motion, TMJ sounds, and impairment of chewing.

**Results:**

Cone beam CT images of the TMJs revealed joint space changes in all patients and degenerative bone changes in 20% (8/40) of the patients. The patients were diagnosed as having disc displacement without reduction with limited opening. Successful reduction of displaced disc had been achieved in the treatment. And pain at rest and pain on mastication had substantially decreased in all patients and mandibular function and mouth opening had significantly improved since 2 weeks’ follow-up. The overall success rate kept at a high level of 97.5% (39/40) at 6 months and 5 years’ follow-up.

**Conclusions:**

The technique combining mandibular condylar movement exercise with auriculotemporal nerve block and dextrose prolotherapy is straightforward to perform, inexpensive and satisfactory to young patients with TMJ closed lock.

## Background

Temporomandibular joint (TMJ) closed lock is a clinical condition that is mostly attributed to anterior or anteromedial disc displacement without reduction (DDwoR). DDwoR can be a quite debilitating intra-articular disorder, causing significant pain and dysfunction that disturbs the patient’s quality of life with the potential for persistence of symptoms and degenerative joint diseases (DJD) [1–8]. The possible mechanism for jaw locking and DDwoR progression has been proposed to begin as a displaced disc obstructing the forward condylar translation, and direct mechanical injury from joint overloading and hypoxia-reperfusion injury would result in release of free radicals into the synovial fluid causing degradation of hyaluronic acid and eventually a vacuum effect (suction cup effect). The end result of these proposed pathological processes leads to anchored disc phenomenon, and degenerative progression in the longer term [1–3].. Recent studies suggest an increasing frequency of degenerative TMJ changes in adolescents and young adults, which is associated with recent-onset TMJ closed lock. Therefore, early interventions are strongly recommended for symptomatic young patients to minimize the possibility of DJD and dento-maxillofacial consequences [8–10].

In view of therapeutic effects, and risks and costs associated with more complex interventions, patients with symptomatic TMJ closed lock should be initially treated by the least invasive intervention focusing at speeding up the process of alleviation of pain and of improvement in mouth opening [1, 3, 11–15]. The most commonly applied methods of closed lock conservative management include: education and counseling, mandibular manipulation, splint therapy, exercise therapy and pharmacotherapy [12–16]. Validity of therapeutic exercise has been mostly supported by studies from the viewpoint of evidence-based medicine. The standard therapy includes self-exercise with or without additional jaw manipulation, cognitive-behavioral therapy, and education for TMD [14–17]. However, the type of exercise and manual technique differ among researchers, and the treatment effect is easily affected by pain level and locking duration. Patients generally need to take analgesic drugs for weeks, perform mandibular condylar exercise and/or wear splints for months. The reduction of signs and symptoms of TMJ closed lock seems to be related more to the passage of time, but not to the exercise. Further, some long-term survey data indicate that temporomandibular dysfunction remains a recurrent or persistent condition in 50% of symptomatic patients diagnosed with TMD even after successful physical therapy [4–7, 18–20].

By contrast, in designing this study we scheduled anesthetic block of auriculotemporal nerve to numb TMJ area before exercise procedures. The regional anesthesia can reduce pain and protective muscle splinting, increase the mandibular range of motion, and assist in providing a more manageable treatment [21, 22]. Afterwards, hypertonic dextrose prolotherapy targeting retro-discal tissues was used to rehabilitate ligaments or tendons that compose the posterior band. The hypothesis were: sufficient exercise could produce stretching forces to overcome the vacuum effect of the displaced disc and result in full reduction of the disc in one treatment, furthermore, strengthening of the posterior band junction could help prevent recurrence of anterior displacement of the disc. Long-term observation was conducted to evaluate the clinical efficacy of the method in the management of TMJ closed lock adolescents and young adults [23, 24].

## Materials and methods

### Patients

Forty patients (16–30 years old) were enrolled between Jun 2014 and Jun 2015. All these patients had undergone conservative treatment before this procedure, but did not respond to the same. All the patients and/or their patients gave their informed consent for the treatment. They were assessed clinically and radiographically by a senior oral surgeon. The clinical diagnosis criteria for TMJ closed lock were: joint clicking followed by sudden onset of pain and limited mouth opening without clicking; joint pain and limitation in jaw opening severe enough to interfere with ability to eat; a painful locking duration over a week; history of recurrent lock. Cone beam computed tomography (CBCT) images of the TMJs were obtained and evaluated in consecutive slices. The exclusion criteria were: less than 16 years of age, inability to understand the treatment; history of psychoses; maxillomandibular developmental abnormality.

### Treatment procedure

**Fig. 1:**
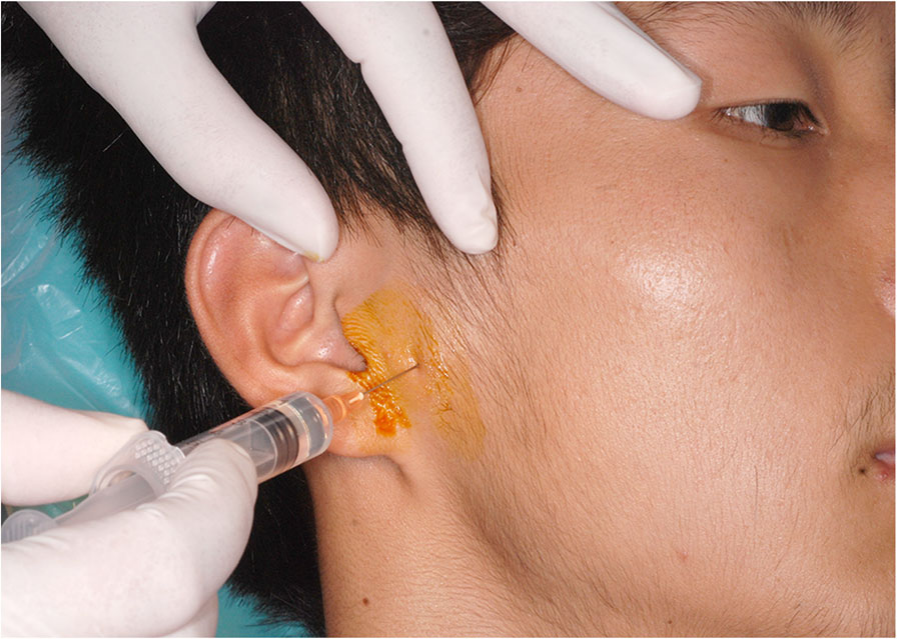
The figure shows the injection point for the patient

Firstly, the patients received anesthetic blockages of the auriculotemporal nerve with injections of 2 ml 2% lidocaine without vasoconstrictor. The injection method was as follows: (1) mandibular condyle was palpated to find the condylar neck for introduction of anesthesia while patients were instructed to open and close the mouth; (2) then the patients were asked to close the mouth and keep in a resting position; (3) a 35-mm-long and 0.5-mm-diameter needle was inserted about 6 mm anterior to the junction of the tragus and lobule and directed to the lateral bone surface of the condylar neck to deposit 0.5 ml lidocaine; (4) the needle was penetrated around the backside of condyle and into the posterior periarticular tissues to a depth of about 25–28 mm where 1.0 ml lidocaine was deposited; (5) the needle was gradually withdrawn meanwhile the final 0.5 ml lidocaine was deposited. Figure 1 shows the injection point for the patient.

**Fig. 2:**
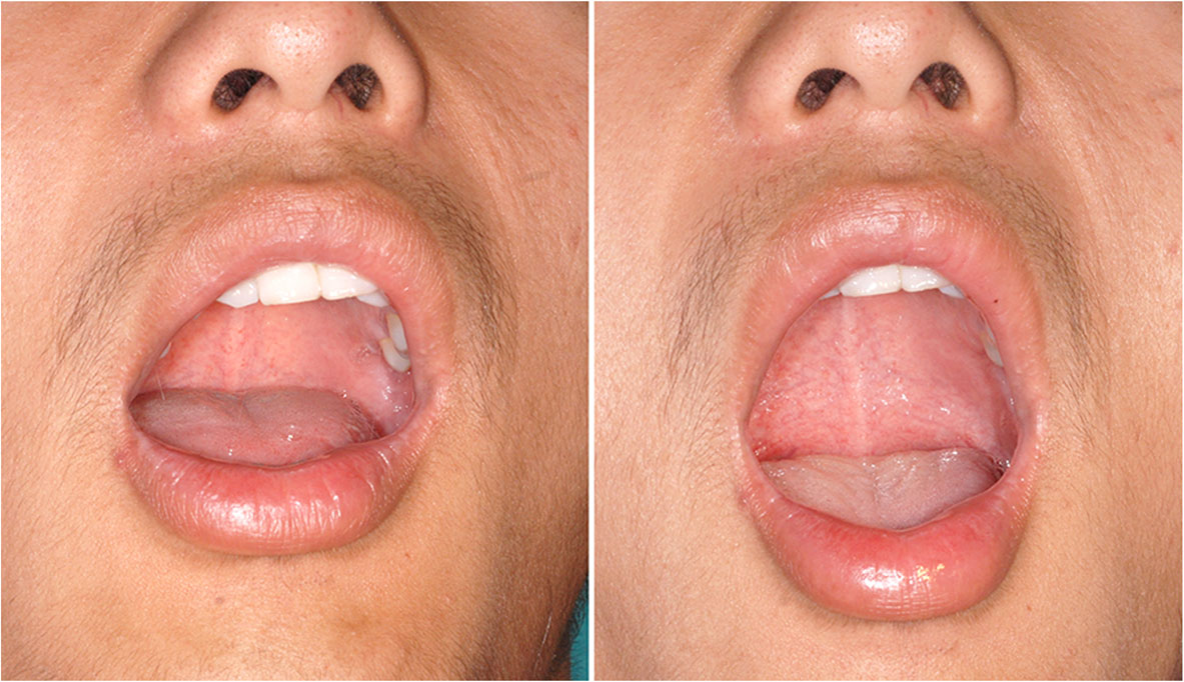
The figures show the maximum mouth opening before and after the mandibular condylar movement exercise

Secondly, the patients were guided to perform mandibular condylar movement exercise to achieve sufficient recovery of the mouth-opening in about 10 min. The exercise consisted of repetitive and incremental mouth opening and bi-lateral movement of the mandibular jaw. Patients could use their fingers to pull down on lower tooth while opening the jaw to the greatest extent, or exercise maximum mouth opening and maximum tongue protrusion. Successful reduction of displaced disc was indicated by returning of joint clicking during mandibular condylar movement exercise, and recovery of mouth opening up to 35-mm width. Figure 2 shows the maximum mouth opening before and after the exercise.

Finally, the patients received injection of 2 ml 50% dextrose as proliferation therapy (prolotherapy) in retro-discal area. Because the auriculotemporal nerve runs around the mandibular condyle’s neck and passes through the retro-discal site of TMJ. The dextrose prolotherapy injection were designed to use the same method as described above in anesthetic blockages of auriculotemporal nerve. Patients were advised to use NSAIDs as well as local application of ice in following days for possible post-treatment pain.

### Analyses

Clinical examinations and questionnaires were completed at baseline and at 2 weeks, 2 months, 6 months follow-ups, and a telephone survey was made at 5 years. The effectiveness of treatment was assessed using outcome measures recommended by the International Association of Oral and Maxillofacial Surgeons (IAOMS). Data collection included pain intensity and frequency, jaw range of motion, TMJ sounds, and mandibular function impairment [11]. All complications during any of the treatments were recorded. Post-treatment CBCT studies of the TMJs were performed at 6 months. Success criteria consisted of the disappearance of or very mild arthralgia, mouth opening of more than 35 mm, and the ability to eat a normal diet [6]. Statistical analysis of the evaluation parameters was performed using Microsoft Excel.

## Results

The initial CBCT image of TMJs revealed joint space changes in all patients and degenerative bone changes in 20% (8/40) of the patients before the treatment. Patients were diagnosed according to the Diagnostic Criteria for Temporomandibular Disorders (DC/TMD) as having disc displacement without reduction with limited opening (Axis I Group IIb), and DDwoR with concomitant degenerative joint disease (Group IIb/IIIb). The clinical characteristics of the study population are given in Table 1.

**Table 1:** The clinical characteristics of the study population

RDC/TMD Axis I Group	Age distribution ≤20 / 21–25 / 26–30	Gender (F/M)	Time since first episode of TMJ close lock (Months, mean ± SD)
Group IIb	10 / 16 / 7	25/7	7.56 ± 8.65
Group IIb/IIIb	2 / 5 /1	8/0	14.14 ± 8.85

Under auriculotemporal nerve block, patients could have their displaced disc reduced by self-exercise in the treatment. Significant improvement of pain at rest and pain on mastication were reported two weeks after the treatment. Dull pain due to injection of hypertonic dextrose was reported and could be alleviated effectively by oral analgesics (0.8 ± 2.3 tablets/per patient) in the first two days. Slight numbness at the injection site was reported by 6/40 patients (15.0%) and recovered within a week.

Analyses of IAOMS recommended outcome variables by time effect are shown in Table 2. The mandibular function and mouth opening basically returned to normal since then and kept in a stable condition from 2 weeks to 6 months follow-up. Only one patient claimed mouth opening limitation (maximum opening 30 mm) and asked for splint therapy at two months follow-up, who was then classified as unsuccessful case. Four patients requested additional injections for further stabilization of the joint at 6 months, and 2 patients requested additional injections in contralateral joint at 6 months and 5 years. The overall success rate kept at a high level of 97.5% (39/40) at 6 months and 5 years’ follow-up.

**Table 2:** Follow-up of IAOMS recommended variables and additional injections

		Base line	2 weeks	2 months	6 months	5 years
Vertical range of motion	< 35 mm	40	5	4	1	1
	≥35 mm	0	35	36	39	39
Lateral and protrusive movement	< 6 mm	27	2	1	1	1
	≥6 mm	13	38	39	39	39
Impaired mandibular function	Yes	40	4	2	1	1
	No	0	36	38	39	39
Excessive joint pain frequency	Yes	40	5	4	1	1
	No	0	35	36	39	39
Excessive joint pain intensity	Yes	40	3	2	1	1
	No	0	37	38	39	39
Joint clicking	Yes	0	0	2	7	7
	No	40	40	38	33	33
Additional injections for enhancement of curative effect	Group IIb		0	0	1	0
	Group IIb/IIIb		0	0	3	0
	Contralateral joint		0	0	1	1

**Fig. 3:**
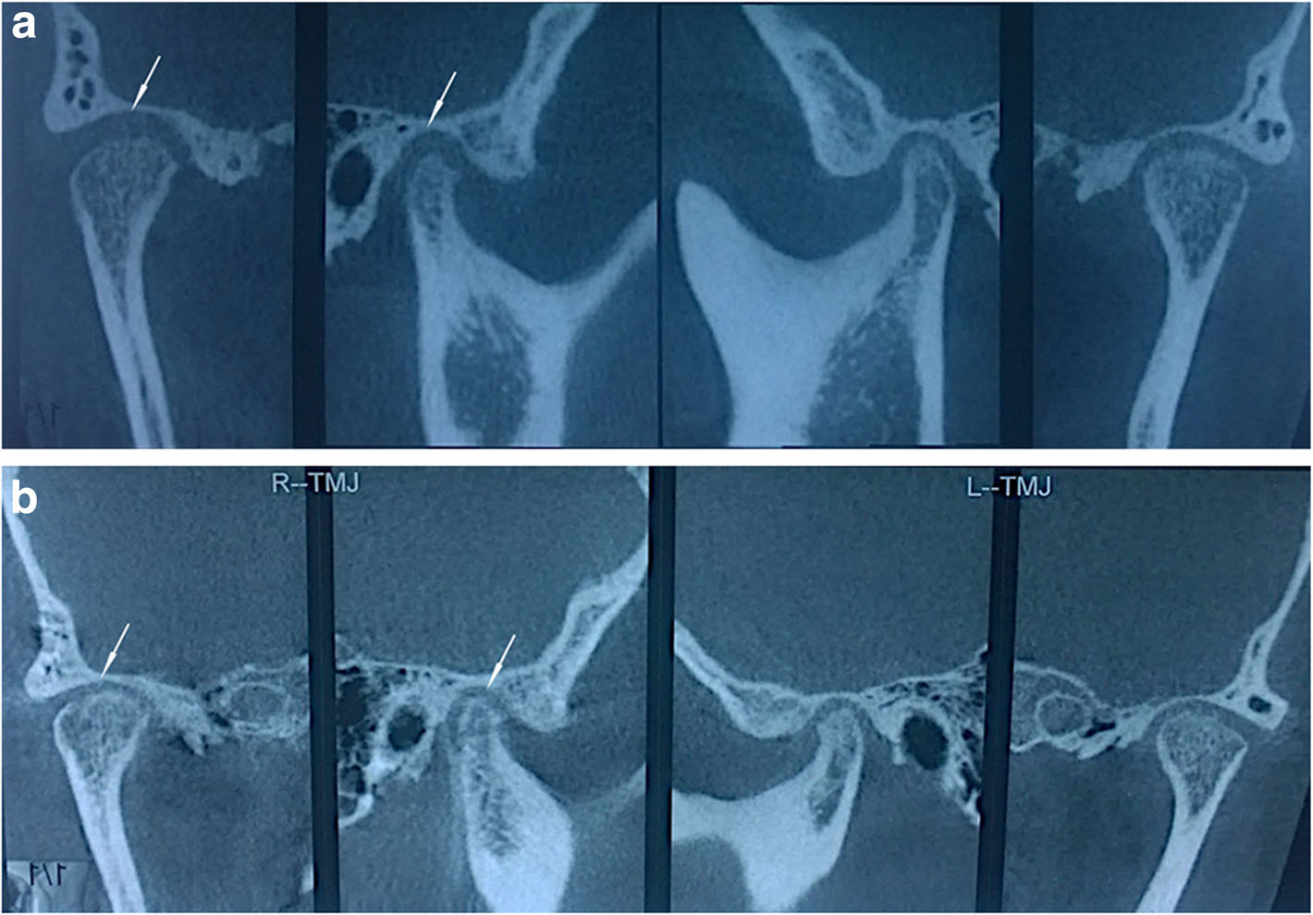
The figures show the pre- (**a**) and post-treatment (**b**) CBCT images of the TMJs

The follow-up CBCT images confirmed more ideal articular fossa-condyle relationship. The dominant location of affected condyles changed from a posterior position to a media position in all patients. Condylar repair was also observed in patients with degenerative joint disease. No progressed condylar bone destruction or condyle deformation were observed. Figure 3 shows the pre- and post-treatment CBCT images of the TMJs.

## Discussion

This study was designed to confirm the clinical efficacy of an alternative method of conservative treatments for TMJ closed lock in adolescents and young adults. We innovatively combined mandibular condylar movement exercise with auriculotemporal nerve block and dextrose prolotherapy to improve curative effects. The technique is unique, to the best of our knowledge, in that TMJ closed lock reduction and reinforced articular disc repositioning could be accomplished in one conservative treatment. The therapeutic process is greatly simplified and shortened.

As the recommendations of the International RDC/TMD Consortium Network (2014), the episode of TMJ “closed lock” has good diagnostic validity for the disc displacement without reduction with limited opening (ie, sensitivity 80%; specificity 97%) [25]. Distinct combination of symptoms could be observed in these patients: previous clicking of the TMJ; limitation of mouth-opening immediately after the joint had stopped clicking; oro-facial pain at rest and at mastication; limitation of lateral movement away from the affected side; and deviation of the mandible to the affected side on opening the mouth [3]. In this study, the symptoms combination has been used as strict inclusion criteria. The enrolled patients all have the typical indications for acute TMJ closed lock. A relief of more severe symptoms can be more persuasive evidence for the curative effects of the treatment. And patients’ willingness to accept therapies that can achieve rapid results are fairly strong.

Lei J, et al. reported that the prevalence of degenerative TMJ changes could be up to 59.30% in chinses adolescents and young adults with recent-onset disc displacement without reduction [9]. Most of these degenerative changes were early-stage osteoarthritic (OA) changes, including loss of continuity of the articular cortex and surface erosion or destruction identified by high-resolution CBCT. Late-stage OA changes (deviation in form and osteophyte) occurred in 13.63% of the symptomatic TMJs. This is basically consistent with the findings of this study, where 20% (8/40) of the DDwoR patients also presented with degenerative joint disease. Adolescents and young adults may be particularly vulnerable to consequences of degenerative joint disease since normal condylar formation could be hindered [26]. Early diagnosis and intervention is thus prudent to improve the possibility of condylar repair and regeneration to restore TMJ form/structure in adolescents/young adults [27].

TMJ closed lock start from “mechanical” joint disorder, in which a displaced disc obstructs the forward condylar translation resulting in restricted mouth opening [3]. Theoretically, mandibular condylar movement exercise could overcome the interference of displaced discs and increase condylar mobility. However, patients’ attempts to increase mouth-opening were frequently hindered by the pain of TMJ. Without enough exercise range, the mandibular condylar movement might displace the disc gradually farther forward to an anterior position. Then the increased mouth opening would be a result of adaptive condylar movement over deformed disc [17]. In fact, a number of studies have reported that disc recapturing by regular exercise is very limited. In more than 75% of the successfully treated cases, discs are still anteriorly displaced and deformed [13, 28]. The betterment of function based on adaptive disc deformity is not a stable condition. There are risks of progression to the more advanced stages by a breakdown in the balance between a patient’s adaptive capacity and overloading of the TMJ. Temporomandibular dysfunction might become a recurrent or persistent condition for a long time [4–7].

Anesthetic block of auriculotemporal nerve can effectively numb TMJ and reduce protective muscle splinting [21, 22]. Subsequently, patients can quickly increase the range of jaw movement through pain-free exercise in several minutes. Meanwhile, sufficient condylar movement produces enough stretching forces on the displaced disc to exceed the anchoring forces produced by vacuum effect, and result in full reduction of the disc. The presence of an audible TMJ click during mandibular condylar movement exercise has been reported by patients in this study, and has been proved an indication for successful TMJ closed-lock reduction [26, 29]. Physical disc reduction in the early course of closed lock is important, because the disc position is not likely to change in long-standing internal derangement [2–4]. Also, an ideal disc-condyle relationship appears extremely important for condylar repair and regeneration in adolescence or young patients with TMJ DJD [26].

When the disc is recaptured through excise therapy, a considerable number of cases will be re-dislocated [14, 26]. We designed hypertonic dextrose prolotherapy to stabilize the repositioning of the disc. Fouda, A.A. has reported that injection of hypertonic dextrose in retro-discal area is effective for reducing clicking and subsequently improving TMJ derangement [23]. Dextrose is considered to be the safest proliferating agent as it is soluble in water, a normal constituent of blood chemistry, and can be injected in large quantities without complications. Hypertonic dextrose solutions at the injection site dehydrate cells, which leads to inflammation of local tissue that in turn triggers the release of growth factors such as fibroblast growth factor, and connective tissue growth factor [30, 31]. The growth factors initiate fibroblast proliferation with production of stronger, thicker, and organized connective tissue [32, 33]. Tissue repair can be evident at 2 weeks with fibrosis and other signs of regeneration at the injection sites [33]. Strengthening of ligaments or tendons that compose the posterior band would prevent anterior displacement of articular disc and also result in a tight feeling in posterior area of affected joint, which would spectacularly diminish without further treatments.

## Conclusions

The technique combining mandibular condylar movement exercise with auriculotemporal nerve block and dextrose prolotherapy is straightforward to perform, inexpensive and satisfactory to young patients with TMJ closed lock. Limitations of this study include a relatively small sample size. Larger and randomized controlled trials are required to further determine whether all TMJ closed lock patients respond similarly to the treatment.

## Data Availability

The data sets supporting the results of this article are included within the article.
